# Characterization of tumor infiltrating Natural Killer cell subset

**DOI:** 10.18632/oncotarget.3453

**Published:** 2015-03-23

**Authors:** Inbar Levi, Hagai Amsalem, Aviram Nissan, Merav Darash-Yahana, Tamar Peretz, Ofer Mandelboim, Jacob Rachmilewitz

**Affiliations:** ^1^ Goldyne Savad Institute of Gene Therapy, Hadassah-Hebrew University Medical Center, Jerusalem, Israel; ^2^ Department of Obstetrics and Gynecology, Hadassah University Hospital-Mount Scopus, Jerusalem, Israel; ^3^ The Surgical Oncology Laboratory, Department of Surgery, Hadassah-Hebrew University Medical Center, Mount Scopus, Jerusalem, Israel; ^4^ Sharett Institute of Oncology, Hadassah-Hebrew University Medical Center, Jerusalem, Israel; ^5^ The Lautenberg Center for General and Tumor Immunology, Institute for Medical Research Israel-Canada, Hebrew University-Hadassah Medical School, Jerusalem, Israel

**Keywords:** NK cells, decidua, VEGF, TILs

## Abstract

The presence of tumor-infiltrating Natural Killer (NK) within a tumor bed may be indicative of an ongoing immune response toward the tumor. However, many studies have shown that an intense NK infiltration, is associated with advanced disease and may even facilitate cancer development. The exact role of the tumor infiltrating NK cells and the correlation between their presence and poor prognosis remains unclear. Interestingly, during pregnancy high numbers of a specific NK subset, CD56^bright^CD16^dim^, are accumulated within first trimester deciduas. These decidual NK (dNK) cells are unique in their gene expression pattern secret angiogenic factors that induce vascular growth. In the present study we demonstrate a significant enrichment of a CD56^brigh^CD16^dim^ NK cells within tumors. These NK cells express several dNK markers including VEGF. Hence, this study adds new insights into the identity of tumor residual NK cells, which has clear implications for the treatment of human cancer.

## INTRODUCTION

Tumor infiltrating Natural Killer (NK) lymphocyte cells, that are part of the innate immune system, have the ability to both lyse cells lacking major-histocompatibility-complex (MHC) proteins and to provide immunoregulatory cytokines [1]. It is well established that NK cells have the ability to recognize and kill tumor cells *in vitro*. However, as opposed to cytotoxic T cells, the *in vivo* function of NK cells against tumors is much less studied. The presence of tumor infiltrating NK may indicate an ongoing immune response toward the tumor. Nevertheless, in many cases there is no correlation between the extent of tumoral NK cell infiltration and a favorable prognosis. On the contrary, many studies have shown that an intense lymphocytic infiltration, NK cells in particular, is associated with advanced disease [2–4] and may even facilitate cancer development. For example, a correlation between high NK concentration in the ascites fluid from ovarian cancer patients and advanced disease was found [5]. The exact role of the tumor infiltrating NK cells as well as the correlation between their presence and prognosis in cancer is still unclear.

In the case of tumor infiltrating lymphocytes (TIL), a solution for the paradox of inverse correlation between the extent of infiltration and favorable prognosis is the recruitment of regulatory lymphocytes to the tumor beds. Curiel et al. [6] have previously demonstrated a specific recruitment of regulatory T cells (Treg) into ovarian cancer tissue. These cells have inhibitory activities toward CD4 helper and CD8 cytotoxic T cells. The authors further established a correlation between the number of tumor-derived Treg cells and patient's survival. Hence, attracting these regulatory cells to tumor beds may represent a mechanism by which tumors may evade the host immune response. Therefore, it is important to also characterize the nature of NK cells within tumor beds.

One of the most intriguing mechanisms of early pregnancy is the maternal immune tolerance toward the semi allogeneic fetus. In contrast to other immune privileged organs such as the retina, in the first trimester decidua there is an abundance of immune cells. In fact about 40% of the total decidual cells are immune cells, mainly lymphocytes of which more than 70% are natural killer cells (7). This is in comparision to peripheral blood where NK cells comprise less than 10%. This outstanding enrichment of NK cells is a unique decidual phenomenon.

Peripheral natural killer (pNK) cells are comprised of two different subsets, the predominant CD56^dim^NK cell subset (about 90 percent) and a much smaller CD56^bright^ NK cell subset [7]. CD56^dim^ pNK cells express high levels of CD16 and both CD94-associated lectin-like NKG2 receptors and killer cell immunoglobulin like receptors (KIRs). These are granular cells that are known to be cytotoxic. In contrast, CD56^bright^ pNK cells are mostly lacking granules, CD16, and KIRs (CD56^bright^CD16^dim^ subset). Interestingly, most decidual NK cells (dNK) are characterized by CD56^bright^CD16^dim^ phenotype, and exhibit low cytotoxic activity [8].

The resemblance of dNK cells to the CD56^bright^ pNK subset, has suggested that dNK cells are derived from CD56^bright^ pNK cells that are seeded in the uterus and undergo further differentiation in the decidual microenvironment [9]. Strominger and his colleagues [8] have previously demonstrated a difference in the gene expression pattern of dNK and the two subsets of pNK cell populations including the CD56^brigh^CD16^dim^ subclass. Significantly, this analysis has revealed multiple genes differentially or uniquely expressed in dNK cells among which are two anti-inflammatory proteins, namely human placental protein 14 (PP14/glycodelin) and galectin-1. In turn, PP14 has been previously shown to suppress the cytotoxic function of NK cells [10].

The reason for abundance of dNK cells in early pregnancy is unknown. Two possible explanations have been proposed for the paradox of attracting large numbers of potentially cytolytic cells to the feto-maternal interface. Decidual NK cells may serve as regulatory (inhibitory) NK cells resembling the role of the Treg cells in tumors thus inhibiting immune responses in the deidua. Another, not mutually exclusive role is that dNK cells might influence maternal mucosal and arterial function and/or placental trophoblast invasion. Activated dNK cells are now considered critical for appropriate endometrial angiogenesis in early implantation site development and in non-gestational endometrium. Indeed, it has been previously demonstrated that dNK cells secrete angiogenic factors that induce vascular growth in the deciduas and can promote tumor growth in animal models [11].

In the present study we hypothesized that similar to deciduas, in solid tumors, CD56^bright^ CD16^dim^NK cells are specifically recruited and accumulate, thus contributing to the tumor development. We characterized tumor infiltrating NK cell subsets and demonstrated that similar to deciduas, there is a significant enrichment of the CD56^brigh^CD16^dim^ subset and that these cells express the pro-angiogenic factor VEGF. Thus, this unique recruited NK subtype may represent cells that participate in the tumor associated-stroma.

## RESULTS

### Infiltrating CD56^+^ NK cells in breast and colon tumor tissues

**Figure 1:**
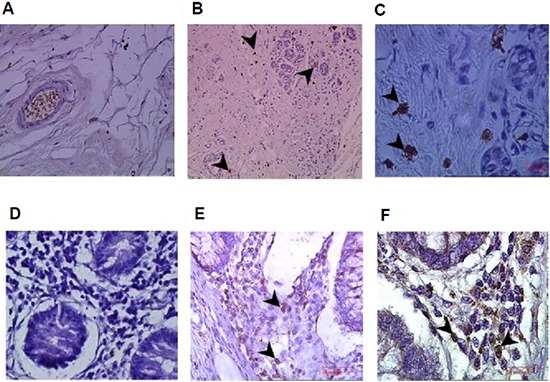
Enrichment of infiltrating CD56+ natural killer (NK) cells in colon and breast tumors Immunohistochemistry staining for CD56 in breast and colon cancer samples and their adjacent normal tissues. **(A)** Normal breast tissue adjacent to the tumor (magnification x400); **(B, C)** Breast tumor tissues demonstrating infiltrating CD56^+^ NK cells (arrows; magnification x200 and x1000, respectively). **(D)** Normal colon tissue adjacent to the tumor (magnification x400); and **(E, F)** colon tumor tissues sections demonstrating infiltrating CD56^+^ NK cells (arrows; magnification x400 x1000, respectively).

Previous studies have described infiltration of lymphocytes and NK in particular into tumors. To confirm the presence of NK cells within breast and colon tumors, *in situ* immunohistochemistry of tumors and peritumoral tissue sections was performed using mouse anti human CD56 mAb. Our results demonstrate a significant enrichment of infiltrating NK cells within the tumor tissues of breast (Figure 1B and 1C) and colon cancer (Figure 1E and 1F), as compared to the adjacent peritumoral breast (Figure 1A) and colon (Figure 1D) tissues where NK cells are absent.

### Selective enrichment of infiltrating CD56^bright^CD16^dim^ NK cells within tumor tissues

**Figure 2:**
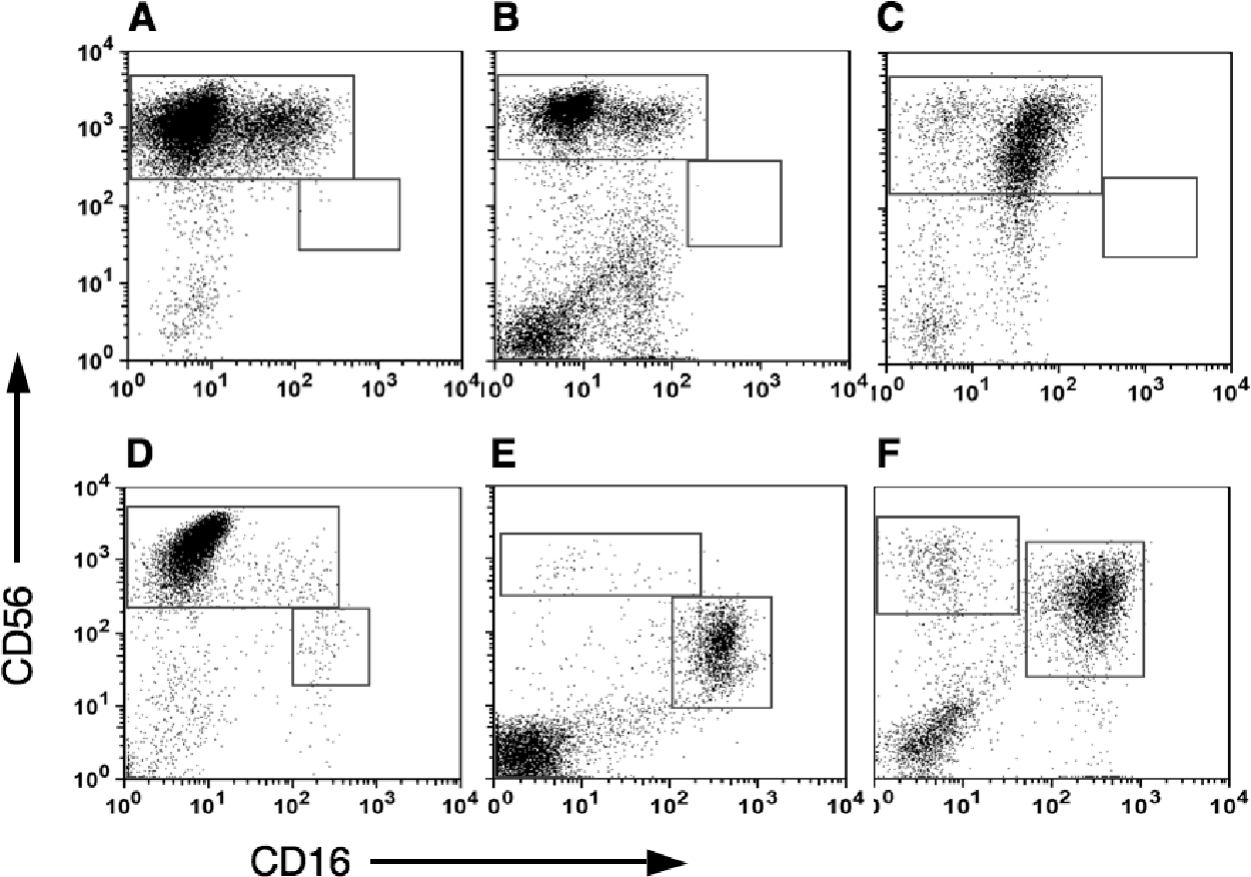
Subset analysis of NK cells in Tumor infiltrating lymphocytes (TILS) Tumor samples were cultured in medium supplemented with recombinant human IL-2 for 2–3 weeks and then the tumor infiltrating lymphocytes were isolated by ficoll gradient centrifugation. NK subset analysis was performed by direct anti-CD3, CD16 and CD56 staining and flow cytometric analysis. CD16 versus CD56 expression of CD3^−^-gated lymphocytes is shown. Representative samples of NK subsets distribution in breast carcinoma **(A)**, Melanoma **(B)** and colon cancer **(C)**, are shown. The tumor NK subset distribution is compared to that seen in decidual lymphocytes **(D)** and PBMC **(E)** as well as IL-2 treated PBMC **(F)**, used as control.

While many studies have demonstrated infiltration of NK cells into tumor tissues using immunohistochemistry staining, these studies failed to characterize the exact subset of infiltrating NK cells. To further characterize tumor infiltrating NK subsets, tumor lymphocytes from breast carcinomas, melanoma and colon cancer tissues were isolated through lympocyte expansion using IL-2. Utilizing multi-color staining and flow cytometric analysis, we identified that the majority of the CD3^−^-gated lymphocytes infiltrating cells belong to the CD56^bright^CD16^dim^ NK subset (Figure 2A–2C). These tumor NK cells resembled deciduas lymphocytes where the majority of NK cells stained as CD56^bright^CD16^dim^ (Figure 2D). This was in sharp contrast to the distribution of NK subsets in PBMC where about 90–95% of the peripheral NK cells express the CD56^dim^CD16^bright^ phenotype and only 5–10% of the NK cells are of the CD56^bright^CD16^dim^ subset (Figure 2E).

**Figure 3:**
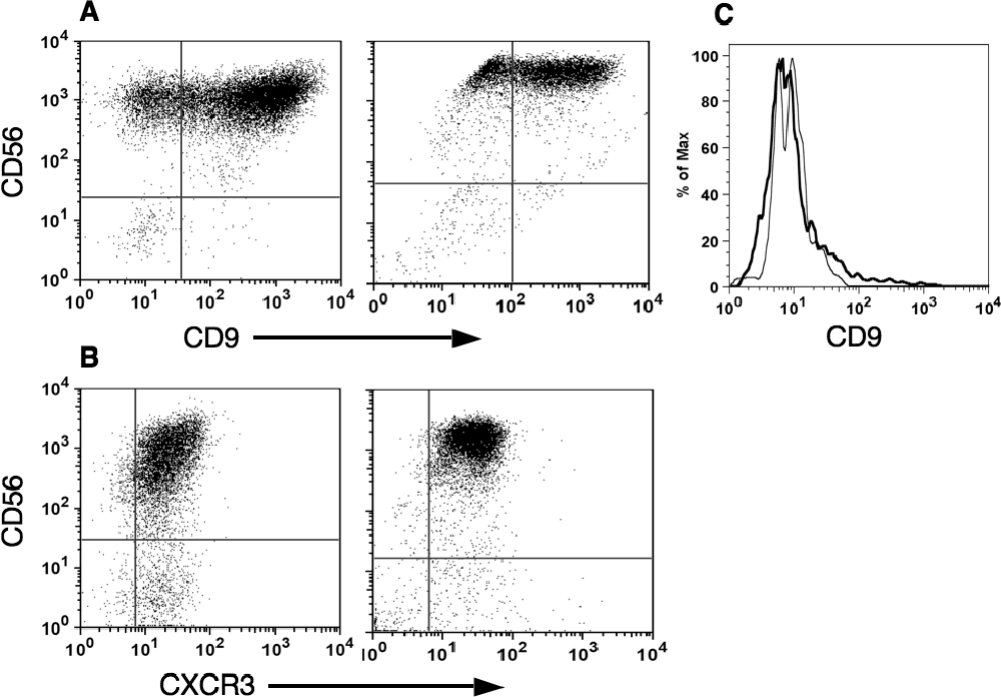
CD56^bright^ natural killer cells express specific dNK surface markers Tumor and decidual infiltrating lymphocytes were isolated as described in Figure 2. The expression of CD56 versus either CD9 **(A)** or CXCR3 **(B)** in CD3^−^-gated cells isolated from either breast tumor (A, left; *n* = 3), colon cancer (B, left; *n* = 3) or deciduas (A and B, right; *n* = 3), is shown. As control for IL-2 treatment, the expression of CD9 in CD3^−^- CD56^high^ gated cells from either PBMC (black line) or IL-2 treated PBMC (grey line), is shown **(C)**.

In addition, tumor infiltrating CD56^bright^NK cells from breast and colon carcinomas highly express CD9 and CXCR3, similar to the reported exclusive expression of these cell surface markers by decidual NK cells as opposed to pNK cells of both subsets [8, 9] (Figure 3).

This subset profile of NK cells in tumors infiltrating lymphocytes is not a consequence of their incubation in IL-2 and selective expansion of CD56^bright^CD16^dim^ subset, as no significant change in the NK subset profile of freshly isolated PBMC (Figure 2E) was observed in PBMC that were cultured in parallel with IL-2 (Figure 2F). Furthermore, culturing PBMC with IL-2 did not significantly change CD9 expression in CD56^bright^NK cells (Figure 3C).

**Figure 4:**
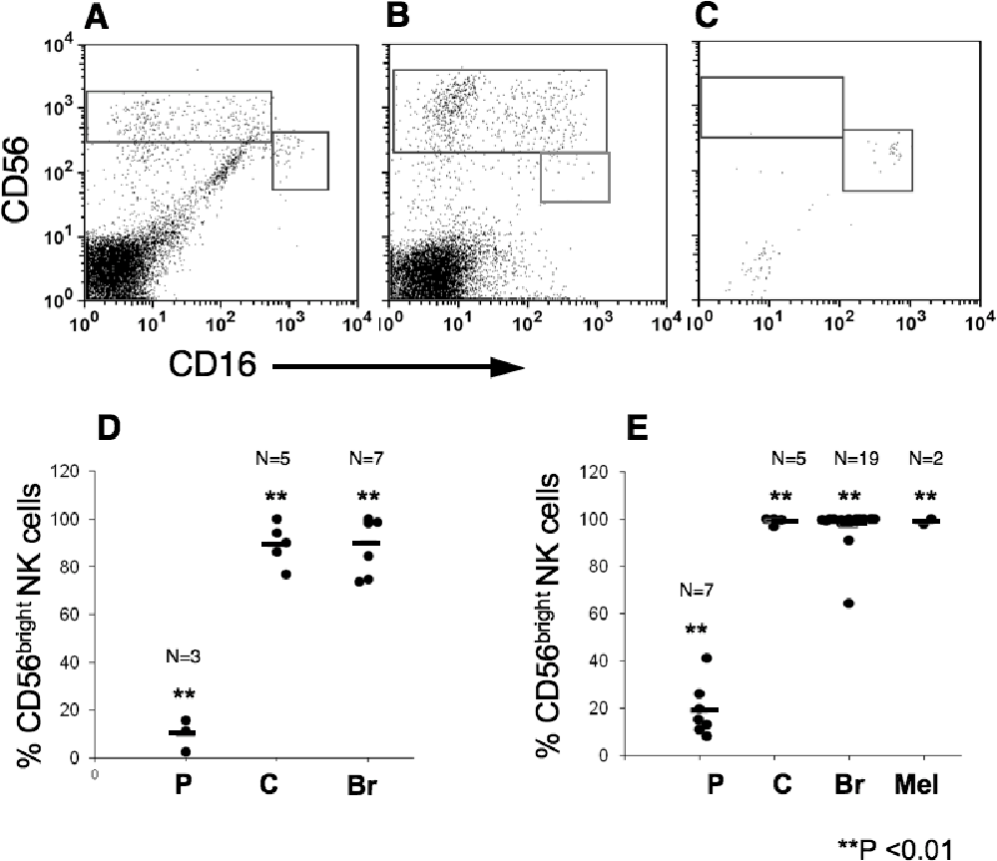
CD56^bright^ CD16^dim^ natural killers subset are highly enriched within melanoma, breast and colon tumors Lymphocytes were isolated from tumor tissues by mechanical dissociation and enzymatic digestion and were then immunostained, as above. CD16 versus CD56 expression of CD3^−^-gated lymphocytes is shown. Representative samples demonstrating the enrichment of CD56^bright^ CD16^dim^ NK subset in breast **(A)**, colon **(B)** but not in Non-small cell lung carcinoma **(C**; *n* = 4) tissues. The mean frequencies of CD56^bright^ CD16^dim^ cells out of total NK cell numbers in lymphocytes isolated either mechanically **(D)** or by IL-2 culturing **(E)** from various tumors. In comparison the percentage of CD56^bright^ CD16^dim^ cells in PBMC (D) or IL-2-treated PBMC (E), is shown. (P), PBMC; (C), colon; (Br) breast; and (Mel) Melanoma tumors. **p < 0.01.

To avoid any effect that either IL-2 or culturing may have on the NK cells we further directly isolated lymphocytes from breast, colon and lung carcinomas. While as expected the overall lymphocyte number was significantly lower, the NK subset profile distribution in breast and colon carcinomas were similar to that obtained in TIL (Figure 4). Statistical analysis confirmed the presence of more than 90% CD56^bright^CD16^dim^ NK subset within the different tumors, compared with around 10% of this subset in pNK (Figure 4B). In contrast to these two carcinomas only few NK cells could be detected in lung carcinoma tissues and most of these NK cells were of the CD56^dim^CD16^bright^ NK subset (Figure 4C).

### Enrichment of infiltrating CD56^bright^CD16^dim^ NK cells within pleural and peritoneal fluids of cancer patients

**Figure 5:**
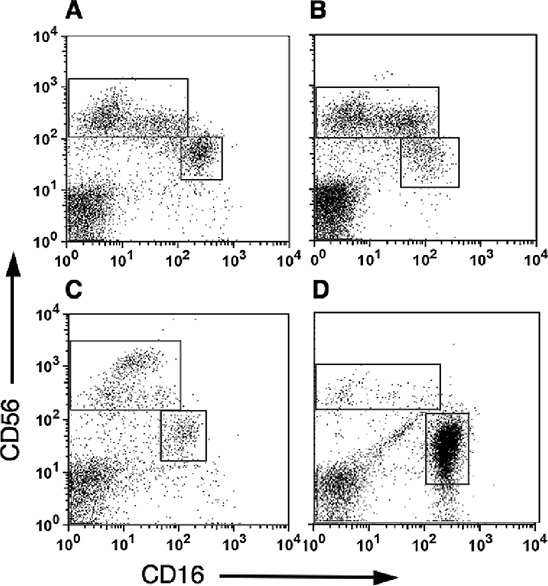
CD56^bright^ CD16^dim^ natural killers cells are highly enriched within pleural and peritoneal fluids from cancer patients Mononuclear cells isolated either from pleural fluids collected from melanoma **(A**; *n* = 3) and breast cancer patients **(B**; *n* = 4), or from peritoneal fluids collected from gastric cancer **(C**; *n* = 1) and pancreatic cancer **(D**; *n* = 1) patients. CD16 versus CD56 expression of CD3^−^-gated lymphocytes is shown.

We further characterized NK subsets in pleural fluids taken from patients with melanoma and breast cancer. The majority of pleural NK cells belong to the CD56^bright^CD16^dim^ subset (Figure 5A–5B), yet the relative enrichment of this subset seems to be lower than that observed in the respective tumor tissues. Similar results were obtained from a peritoneal fluid sample collected from a patient with gastric cancer (Figure 5C). It is interesting to note, a sample of peritoneal fluid from a patient with pancreatic cancer had significantly higher numbers of the CD56^dim^CD16^bright^ subset resembling that of pNK (Figure 5D).

Taken together, the data clearly demonstrate that at least in melanoma, colon and breast carcinoma there is a clear and significant enrichment of the CD56^bright^CD16^dim^ subset of NK cells. This appears to be specific to certain tumors but not all as evideced in the case of lung cancer.

### Breast and colon tumor infiltrating CD56^bright^NK cells express the pro-angiogenetic factor VEGF

Recently a significant shift occurred in the way we understand dNK cell functions. dNK cells are now considered critical for appropriate endometrial angiogenesis in early implantation site development, as it has been demonstrated that dNK cells secrete angiogenic factors that induce vascular growth in the deciduas and can promote tumor growth in animal models [11]. This suggests that NK cells may also have pro-tumorigenic functions through the promotion of angiogenesis.

**Figure 6:**
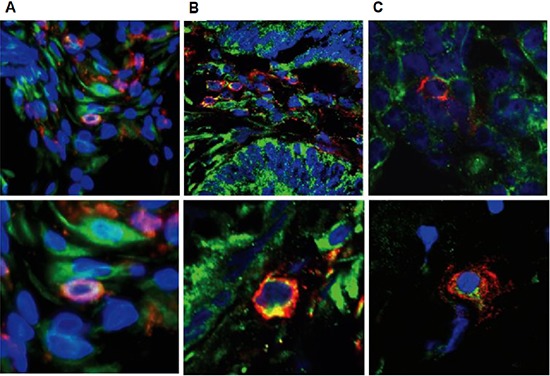
Tumor infiltrating NK cells express VEGF Confocal microscopy of representative double immunofluorescence staining for CD56 (red) and VEGF (green) expression in decidua **(A)**, colon tumor **(B)** and breast tumor **(C)** tissues (original magnification x600). Blue: nuclear staining using DAPI.

Therefore, we subsequently tested whether tumor-infiltrating NK cells express the pro-angiogenic factor VEGF. To that end, sections from colon and breast carcinomas as well as deciduas, used as positive controls, were double immunostained with anti-CD56 and antipan-VEGF antibodies. As shown in Figure 6, the majority of the CD56-positive cells, marked by a red fluorescence on the cell membrane, were positive for VEGF, represented as a green fluorescence, which was essentially localized in the cell cytoplasm, in both colon and breast tumor tissues and as expected, also in deciduas. In contrast, adjacent normal colon and breast tissues were negative for CD56 and VEGF staining (data not shown). Immunostaining for placental growth factor (PLGF) was negative in all samples (data not shown). Unfortunately, we were unable to carry out functional assays using isolated tumor infiltrating NK cells given the limited amount of available tissue and low numbers of NK cells.

## DISCUSSION

NK cells are effector cells of the innate immune system that are known for their ability to kill virally infected, transformed and stressed cells. However, recent studies have revealed a more complex depiction of NK biology and demonstrated that NK cells exert broad and complicated functions that in the past were attributed to the adaptive immune system [12]. Moreover, this blurring of previous distinctions between innate and adaptive immunity is surpassed by the acknowledgment that NK in the human deciduas (namely the CD56^bright^CD16^dim^ subset) seem to have a constructive role given that they take part in regulating the reproductive process.

While NK cells have the ability to kill tumor cells, there is an ambiguity in the literature in regards to the correlation between the presence and number of infiltrating NK cells in cancer and patient prognosis [2, 4]. This raises the question of whether infiltrating NK cells indeed represents an ongoing anti-tumor immunity, as was previously thought, or alternatively NK cells may have other more constructive functions and even facilitate cancer development, reminiscent of the situation in the decidua.

Based on this hypothesis, in this study we have tested whether tumor-infiltrating NK cells resemble dNK cells in respect to their subset characterization and specific markers. Our data clearly demonstrates that the majority of NK cells infiltrating into the three types of tumors tested here (namely melanoma, breast and colon carcinoma) are of the CD56^bright^ subset. This high enrichment of CD56^bright^ subset in tumor infiltrate is in sharp contrast to peripheral blood where this subset represents only 5–10% of the NK cells. Surprisingly, in the non-small-cell lung cancer the overall number of NK cells was very low and there was no significant enrichment of the CD56^bright^ cell subset. This latter finding is in sharp contrast to a previous study by Carrega et al. [13] that has shown consistent enrichment in the CD56^bright^ subset in the tumors, although the mean percentage of CD56^bright^ cells was significantly lower (less than 40 percent) than that seen in deciduas or the 80–90% observed in the three tumor types tested in our study.

Previous studies have established that dNK cells preferentially express high levels of the chemokine receptors CXCR4 and CXCR3 [9, 14]. These receptors are probably involved in the migration of CD56^bright^ pNK cells to the deciduas and their specific accumulation in the fetal-maternal interface. In addition, gene array analysis revealed a set of genes that were preferentially expressed by dNK cells and others were unique to dNK cells and were not expressed by the two pNK subsets [8]. These genes included the cell-surface protein CD9. Interestingly, tumor infiltrating CD56^bright^ NK cells express high levels of two of these dNK markers: CXCR3 and CD9. Tumor infiltrating NK cells may represent a differentially recruited CD56^bright^ pNK cells that acquire a unique phenotype as a result of their interaction with the tumor microenvironment. Their similarity to dNK cells may reflect general effects common to the two different tissues, both of which induce similar regulatory functions.

This specific enrichment of CD56^bright^ NK cells is not limited to the tumor bed itself as it was also observed in the pleural fluids of cancer patients. This enrichment seems to be specific for cancer patients as Pokkali and collegues study has demonstrated that in a mixed population of patients suffering from various diseases in which excess fluid accumulate between the two pleural layers, there was only a small increase in CD56^bright^ NK cells in pleural fluids in comparison with their respective PBMC values [15]. These cases included patients suffering from heart failure, liver cirrhosis, renal failure as well as cancer. Based on our results with individual patients suffering from cancer, the enrichment of the CD56^bright^ may result from the cancer patients' samples. This enrichment of immune cells associated with malignant pleural or peritoneal fluids is reminiscent of the increase numbers of Tregs in cancer patients [16, 17].

In the maternal-fetal interface, there is an unexpected enrichment of lymphocyte cells, while 50–70% of them are NK cells. Hanna et al. [11] have provided evidence that dNK cells regulate trophoblasts invasion and produce pro-angiogenesis factors, such as the VEGF and PLGF and promote angiogenesis and the generation of spiral arteries in the decidua. The parallel between the CD56^bright^ tumor infiltrating NK cells and dNK cells may imply a similar role. Therefore, we examined the expression of pro-angiogenesis factors in tumor CD56^+^NK cells. Double immunofluorescence histochemistry staining has confirmed that CD56^+^NK cells within breast and colon tumors express cytoplasmic VEGF, but not PLGF.

Taken together, the similarities between tumor infiltrating NK cells and dNK cells may reflect a similar maturation of pNK cells (most likely the CD56^bright^ subset) in the decidual and in the tumor microenvironments, a maturation that culminates in distinct regulatory role for this NK population rather than the cytotoxic functions. The present study reveals that a significant effort is still required to understand how to deploy possible NK-based therapies to cure cancer in humans.

## METHODS

### Immunohistochemistry

Tumors and adjacent tissues isolated from surgically resected specimens were fixed in 4% paraformaldehyde for 1 hour and then embedded in paraffin. 5 μm-thick sections were deparaffinized in xylene and rehydrated through a gradient series of ethanol in PBS. Endogenous peroxidase activity was blocked by incubation of the sections in 3% hydrogen peroxide in methanol for 40 minutes. Heat-induced antigen retrieval was carried out using 10 minute incubation at 37°C in PBS containing Trypsin 0.125% and subsequently all sections were blocked with 10% donkey serum (Dako protein blocking solution; Dako, Carpinteria, CA, USA). Next, tissue sections were incubated with primary monoclonal mouse anti human CD56 antibody (Dako) for 1 hour at room temperature followed by 1 hour biotinylated goat anti-mouse IgG secondary antibody incubation (Dako). Streptavidin-HRP was added to each tissue section followed by 0.075% (weight/volume) 3, 3-diaminobenzidinein PBS containing 0.002% (volume/volume) H_2_O_2_ application, yielding a brown color reaction at target antigen sites (Vector Laboratories, Burlingame, California). Finally, all slides were counterstained with hematoxylin (Sigma) to visualize the nuclei and slides were dehydrated in an ascending ethanol series, cleared in xylene, and mounted with Permount Media (Fisher Scientific, Fair Lawn, New Jersey). Appropriate negative controls were derived through substituting the primary antibody with isotype mouse IgG. Evaluation of the immunohistochemical staining was performed by light microscopy using 10 ×, 20 × and 40 × objective lenses.

### Mononuclear cell isolation

Peripheral blood mononuclear cells (PBMC) from the venous blood of healthy donors and from pleural and peritoneal fluids collected from cancer patients were purified by density gradient centrifugation using Ficoll-Histopaque (Sigma Aldrich, St Louis, MI, US).

### Direct *ex-vivo* isolation of tumor and decidua infiltrating lymphocytes

Decidual samples from patients undergoing elective termination of pregnancy in the first trimester between 8 and 12 weeks of gestation and the indicated tumors were collected at the Hadassah Medical Center under approval of Hadassah Medical Center Helsinki Ethics Committee following an informed consent for both deciduas and tumor tissues. Decidua and tumor tissues were washed extensively in PBS before mincing with sterile scissors. Lymphocytes were released from the mechanically dissociated tissue by repeated digestion in RPMI 1640 medium containing 0.1% collagenase type IV (Worthington Biochemical, Lakewood, NJ, USA) and 0.01% DNase I (Roche Diagnostics, Mannheim, Germany) followed by centrifugations and supernatant collection. Single-cell suspensions were filtered through a 70-μm-cell strainer (BD Biosciences, Bedford, USA). The decidua and tumor lymphocytes were separated from other residing cells by ficoll gradient centrifugation, as was previously described [9]. For subsets analysis, each sample was immunostained and tested by flow cytometric analysis as described below.

### Tumor lymphocytes expansion

Each tumor specimen was dissected free of surrounding normal tissue and necrotic areas. Small chunks of tumor (usually 8–16) measuring about 1 to 2 mm were cut with a sharp scalpel from different areas within the tumor specimen. A single tumor fragment was placed in each well of a 24-well tissue culture plate with 2 ml of complete medium (CM) plus 6000 IU/ml of rhIL-2 (Chiron Corp., Emeryville, CA). CM consisted of RPMI 1640, 25 mmol/L HEPES pH 7.2, 100 U/ml penicillin, 100 μg/ml streptomycin, 2 mmol/l-glutamine, and 5.5 × 10 − 5 mol/L β-mercaptoethanol, supplemented with 10% human serum. The plates were placed in a humidified 37°C incubator with 5% CO2 and cultured until lymphocyte growth was evident. Each fragment was inspected about every other day using a low-power inverted microscope to monitor the extrusion and proliferation of lymphocytes. Regardless if lymphocyte growth was visible, half of the medium was replaced in all wells no longer than 1 week after culture initiation. Typically, about 1 to 2 weeks after culture initiation, a dense lymphocytic carpet would cover a portion of the plate surrounding each fragment. When a well became almost confluent, the contents were mixed vigorously, split into two duplicated wells and filled with 2 ml CM supplemented with 6000 IU/ml rhIL-2. Subsequently, the cultures were split to maintain a cell density of 0.8–1.6 × 10^6^ cells/ml, or half of the media was replaced at least twice a week. Each initial well was considered to be an independent TIL culture and maintained separately from the others.

### Flow cytometry

The expression of surface markers was measured by direct immunofluorescence, using either FITC-conjugated CD16, APC-conjugated CD3 (OKT3, eBiosciences Inc. San Diego, US) and PE-conjugated CD56 (BD Biosciences, San Jose, US); FITC-conjugated CD9 (eBiosciences Inc.), APC-conjugated CD3 and PE-conjugated CD56; or FITC-conjugated CXCR3 (R&D systems Inc, Minneapolis), PE-conjugated CD56 and APC-conjugated CD3 antibodies. The immunostained cells (1×10^5^ cells per sample) were analyzed on a FACSCalibur flow cytometer (Becton Dickinson, San Jose, CA, USA) using Cell Quest software.

### Immunofluorescence histochemistry

Frozen breast and colon tumors, adjacent tissues and deciduas tissues were cryostat sectioned. For double labeling, tissues were fixed with 4% PFA and blocked with 10% normal goat serum (NGS; Millipore, Temecula, CA). Slides were incubated for 1.5 hr at room temperature with an anti-CD56 monoclonal antibody (Dako, Carpinteria, CA, USA) diluted 1:100 in 10% NGS followed by three times PBSx1 washes and secondary goat anti-mouse IgG coupled to Cy3 antibody (Jackson ImmunoResearch laboratories Inc, West Grove, PA, USA), diluted in 10% NGS, for 1 hr incubation. Slides were repeatedly washed and then incubated with an anti-VEGF (Vascular endothelial growth factor, A-G isoforms) polyclonal antibody (Millipore) diluted 1:500 in 0.1% triton X-100 (BDH Laboratory suppliers, England) for overnight incubation followed by three times PBSx1 washes and secondary DyLightTM488 conjugated goat anti-rabbit IgG(H+L) antibody (Jackson labs Inc.) diluted 1:100 in 0.1% triton X-100, for 1 hr incubation. Samples were extensively rinsed in PBSx1. For fluorescence preserving, samples were mounted with DAPI containing mounting medium (Vector labs Inc Bulingame, CA). For each tissue type, negative control was carried out by using isotype Ab control. For each tumor tissue, adjacent tissue was also stained and analyzed as negative control. Fluorescence images were viewed and taken on IX81 Motorized inverted confocal microscope equipped with an Ultra-precise Z-axis motor with 10nm step size (Olympus America Inc, NY, USA).

### Statistical analysis

Statistical analyses were performed using Microsoft excel 2004 for Mac, version 11.5.5. The significance was evaluated by paired student *t-*test.

## Abbreviations

NK: Natural Killer cells
dNK: decidual NK
pNK: Peripheral natural killer
PBMC: Peripheral blood mononuclear cells
PLGF: placental growth factor
TIL: tumor infiltrating lymphocytes
VEGF: vascular endothelial growth factor
